# The Prevalence and Characteristics of Exocrine Pancreatic Insufficiency in Patients with Type 2 Diabetes: A Systematic Review and Meta-Analysis

**DOI:** 10.1155/2022/7764963

**Published:** 2022-07-19

**Authors:** Jun Zhang, Jiaying Hou, Dechen Liu, Yingqi Lv, Chi Zhang, Xianghui Su, Ling Li

**Affiliations:** ^1^Department of Endocrinology, Zhongda Hospital, School of Medicine, Southeast University, No. 87 Ding Jia Qiao, Nanjing 210009, Jiangsu, China; ^2^Institute of Glucose and Lipid Metabolism, Southeast University, Nanjing, China; ^3^Department of Endocrinology, First Affiliated Hospital of Xinjiang Medical University, Changji Branch, Changji 831100, Xinjiang, China; ^4^Department of Endocrinology, Hunan Provincial People's Hospital, First Affiliated Hospital of Hunan Normal University, Changsha 410005, Hunan, China

## Abstract

**Background:**

Exocrine pancreatic insufficiency (EPI) is common in patients with type 2 diabetes. However, the prevalence of EPI varies significantly in different studies. Untreated EPI in these patients can adversely affect their nutrition and metabolism. The aim of this study is to estimate the pooled prevalence of EPI in patients with type 2 diabetes and to explore the potential risk factors.

**Methods:**

A systematic search was performed in PubMed, Web of Science, and Embase, which included studies meeting inclusion criteria from 1960 to 1 April 2022. Relevant articles were searched using the combination of Medical Subject Heading (MeSH) terms of “Type 2 diabetes” and “pancreatic exocrine insufficiency.” The Stata 16.0 software was used for data analyses. The random-effects model was used to estimate the pooled prevalence rates and 95% CI using “metaprop program.”

**Results:**

The pooled prevalence of EPI was 22% (95% CI: 15%–31%) in patients with type 2 diabetes and 8% (95% CI: 4%–14%) of them developed severe pancreatic insufficiency. In the subgroup analyses, the prevalence of EPI in type 2 diabetes was correlated with geographic location. The prevalence in Asian countries (35%, 95% CI: 22%–49%) is higher than in Europe (18%, 95% CI: 10%–29%) and Australia (9%, 95% CI: 4%–16%). Furthermore, patients with higher insulin requirements, who are more likely to be insulin-deficient, have a higher prevalence of EPI. The pooled prevalence was 27% (95% CI: 17%–37%) in type 2 diabetes with higher insulin requirement (1 group) and 15% (95% CI: 1%–40%) in patients with lower insulin requirement (2 group). In addition, the morbidity of severe EPI in the higher insulin requirement group (12%, 95% CI: 7%–19%) was sextuple as much as the lower insulin requirement group (2%, 95% CI: 0%–13%). EPI was more common in subjects younger than 60 compared with elderlies (25% vs. 19%).

**Conclusion:**

The prevalence of EPI in type 2 diabetes may be overestimated. Furthermore, the higher prevalence may be closely related to *β*-cell function. Endocrine disease therapy would potentially represent a novel therapeutic approach for patients with type 2 diabetes and EPI.

## 1. Introduction

Diabetes mellitus is a chronic abnormal metabolic condition, which is caused by the dysfunction of endocrine functions of the pancreas islets. The pancreas contains both exocrine and endocrine parts, and the functions of the two parts have been reported to interact with each other. The exocrine pancreatic disease can lead to diabetes of the exocrine pancreas (DEP), which is the second most common type of new-onset diabetes in adults (surpassing type 1 diabetes) [1]. On the other side, exocrine pancreatic dysfunction may occur in patients with diabetes.

EPI is a state in which pancreatic enzyme activity is below the threshold required to maintain normal digestion, resulting in insufficient digestion of nutrients, especially fats [2, 3]. EPI is frequently observed in diabetes mellitus, but the prevalence of EPI is very heterogenous in different studies, especially in patients with type 2 diabetes. The prevalence ranged from 5.1% to 81.8% [4–6]. Most of these studies did not exclude cases with previous pancreatic disease. It is acknowledged that exocrine pancreatic dysfunction also often develops in patients with diseases of the exocrine pancreas [7, 8]. This suggests that the morbidity in earlier studies was possibly inflated, at least in part. In addition, little is known about the clinical characteristics of exocrine pancreatic insufficiency and its optimal treatment. To obtain more realistic results and guide clinical practice, we conducted a systematic review of studies reporting on exocrine insufficiency in type 2 diabetes, limiting inclusion criteria and exclusion criteria. Furthermore, we performed a study-level meta-analysis to explore the prevalence of pancreatic exocrine insufficiency and the related factors.

## 2. Methods

The Preferred Reporting Items for Systematic Reviews and Meta-Analyses (PRISMA) [9] was used to guide study selection, and we prospectively registered in the PROSPERO registry (CRD42021233175).

### 2.1. Literature Search

Searches were conducted in PubMed, Web of Science, and Embase. The search included reports from 1960 to 1 April 2022. Identified studies by our search strategies were imported into Endnote X9 (Thomson Reuters, Philadelphia, PA, USA) software and managed. The searches were restricted to English language and human studies.

### 2.2. Search Strategy

We used the following combination of MeSH terms and text words in PubMed: (“Diabetes Mellitus Type 2”) and (“Exocrine Pancreatic Insufficiency” OR “pancreatic dysfunct^*∗*^” OR “pancreas dysfunct^*∗*^” OR “pancreatic insufficien^*∗*^” OR “pancreas insufficien^*∗*^” OR “Pancreatic elastase-1” OR “coefficient of fat absorption” OR “steatorrhea” OR “exocrine pancreas” OR “exocrine” OR “pancreatic enzyme replacement therapy”). Articles identified were further screened according to the following criteria.

Inclusion criteria: studies in patients with type 2 diabetes; diagnostic laboratory testing for pancreatic exocrine insufficiency was reported; incidence rate or raw data to calculate the rates was reported.

Exclusion criteria: studies in patients with pancreatitis, animal studies, unpublished studies, conference abstracts, or case reports.

### 2.3. Selection of Studies and Data Extraction

Study selection and data extraction were conducted independently by Z. J. and H. J. Y. with any discrepancies reviewed by D. C. L. The abstracts were first reviewed for relevance, and then full-text articles were obtained for further review. References in the included studies were also reviewed for eligibility. The following data were extracted from the included studies: first author, year of publication, region, hemoglobin (HbAlc), diabetes duration, total study population, age, sex, body mass index (BMI), prevalence (number or estimation), and rate of insulin use.

### 2.4. Quality Assessment

The Joanna Briggs Institute Prevalence Critical Appraisal Tool was used to evaluate the quality of selected articles. The tool contains 10 questions that evaluate the confounding, selection bias, bias related to measurement, and data-analysis of included studies. Each question was scored 0 or 1, based on a ‘yes' or ‘no' answer to the questions. The quality of the study was defined as “high risk” if with a total score of 0 and 3, while 4–6 represents a moderate risk and ≥7 was considered a low risk of bias.

### 2.5. Statistical Analyses

Metaprop program [10] in Stata 16.0 was used to estimate the pooled prevalence and corresponding 95% confidence interval (CI). Heterogeneity between the studies was assessed using the I^2^ metric with cutoffs of 25%, 50%, and 75% to define low, moderate, and high heterogeneity, respectively [11]. A random-effects model was adopted since a high level of heterogeneity was identified among included studies [12].

Sensitivity and subgroup analyses were performed to explore possible sources of heterogeneity. Publication bias was tested using Egger's and Begg's test and visual inspection of the funnel plot [13]. *P* < 0.05 was considered as statistical significance.

## 3. Results

### 3.1. Study Characteristics

**Figure 1:**
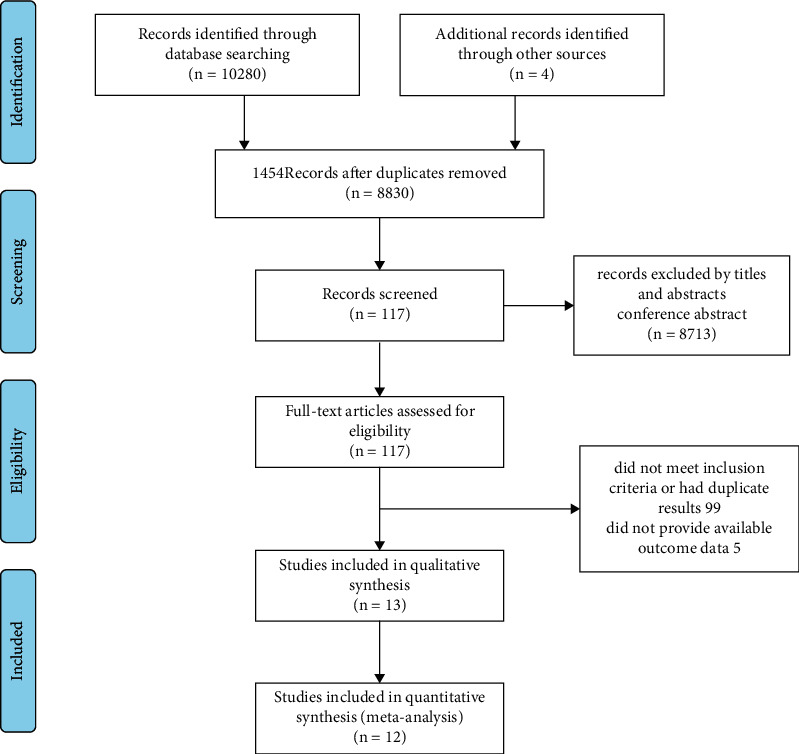
Flowchart for study inclusion.

A total of 10280 studies with potential relevance were identified in the primary search. After removal of duplicates and initial screening of titles and abstracts, full texts of the 117 remaining articles were reviewed. After systematic review, 13 [6, 14–25] studies were included in the final analyses (Figure 1). A total of 2078 patients were identified in the 13 included studies. The characteristics of the included studies and the extracted information are summarized in Table 1. It represents the characteristics of the included studies. A total of 2078 patients with type 2 diabetes participated in these studies, of which 592 patients developed EPI. The pancreatic exocrine function was assessed with the fecal elastase-1 (FE-1) test and 72-hours fecal fat assay. Studies were published between 2003 and 2021. Of them, seven studies were from Europe [14, 16, 20] [19–22], five studies were from Asia [5, 15, 18, 24, 25] and one study was from Australia [23].

### 3.2. Quality Assessment and Risk of Bias

The Joanna Briggs Institute Prevalence Critical Appraisal Tool was applied to each of the studies (Supplementary Table 1). Among the 13 studies included, none were scored 4 or less. None of the studies were considered as low quality. According to sensitivity analyses, we found that one of the study [6] had strong heterogeneity. Visual inspection of the funnel plot suggests an asymmetrical distribution of articles. Removal of this study resulted in a significant change in the effect size of the meta-analysis composite, so we excluded it. No evidence of funnel plot asymmetry was found for Egger's test and Begg's test, indicating a lack of publication bias (*P*=0.837, *P*=0.466).

### 3.3. Meta-Analysis and Data Synthesis

The pooled prevalence of EPI was 22% in T2DM patients, and 8% of them developed severe pancreatic insufficiency. In the subgroup analyses, the prevalence of EPI in type 2 diabetes was correlated to geographic location and insulin use. EPI was more common in subjects younger than 60 compared with elderlies.

### 3.4. The Prevalence of EPI

**Figure 2:**
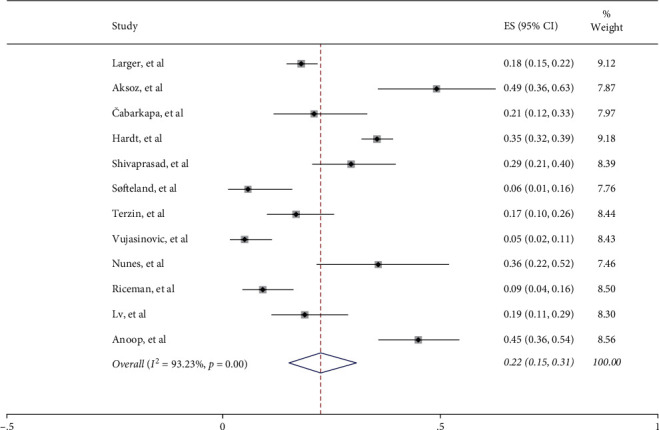
The prevalence of EPI.

The prevalence of EPI in type 2 diabetes was assessed with the random-effects model, and the pooled incidence was 22% (95% CI 15%–31%) (Figure 2). However, high statistical heterogeneity was noticed (I^2^ = 93.23%, *P* < 0.001). Sensitivity analysis was performed. The sensitivity analysis after the removal of each study did not result in significant changes. Therefore, the potential sources of high statistical heterogeneity were explored by further subgroup analysis.

### 3.5. Geographic Distribution and EPI

**Figure 3:**
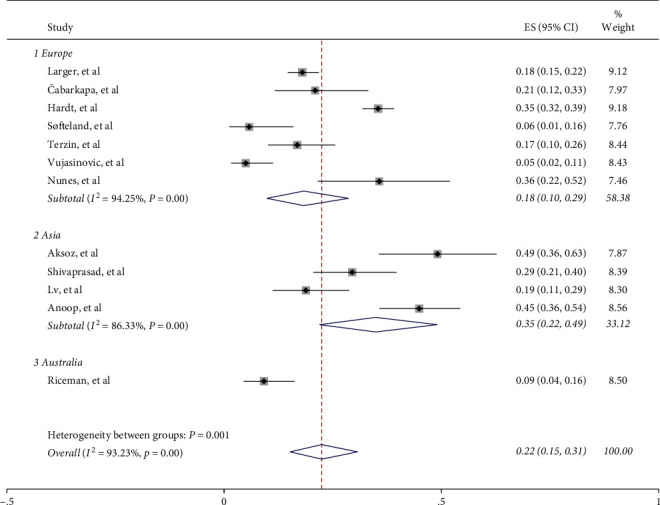
Geographic distribution of EPI.

In the subgroup analyses based on regions, the prevalence of EPI in Asian countries (35%, 95% CI: 22%–49%, I^2^ = 86.33%) is much higher than that in Europe (18%, 95% CI: 10%–29%, I^2^ = 94.25%) and Australia (9%, 95% CI: 4%–16%) (Figure 3). Heterogeneity remained present despite subgroup analysis. Therefore, random-effects models were used to calculate the total effect and subgroup effect. In addition, more rigorous studies are needed to confirm the relationship between geographical location and the prevalence of EPI in type 2 diabetes.

### 3.6. Insulin Use and EPI

A total of 8 studies reported the treatment of insulin in patients with type 2 diabetes, with 1672 participants (84% of all). However, information on antidiabetic treatment other than insulin and the correlation between BMI and daily dose of insulin was not reported. We divided the study populations into the higher insulin requirement group and lower insulin requirement group based on the proportion of patients treated with insulin. 1389 patients (83%) were in the higher insulin requirement group and 283 patients (17%) in the lower insulin requirement group. The pooled prevalence of EPI was 27% (95% CI: 17%–37%, I^2^ = 90.95%) in type 2 diabetes patients with higher insulin requirement group (1 group), while patients with lower insulin requirement group (2 group) had a lower incidence of EPI (15%, 95% CI: 1%–40%, I^2^ = 95.17%) (Supplementary Figure 1).

### 3.7. The Prevalence of Severe EPI

**Figure 4:**
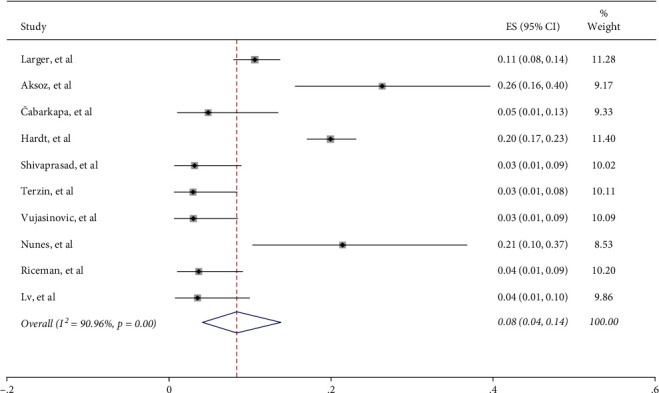
The prevalence of severe EPI.

Most of the included studies categorized exocrine impairment into different degrees, except two [22, 24]. Patients with FE-1 levels of >200 *μ*g/g are considered normal, between 100 and 200 *μ*g/g as mild to moderate EPI, and <100 *μ*g/g as severe EPI [26]. Among the 1820 patients with type 2 diabetes, mild to moderate and severe EPI cases were both reported in 232 patients. After pooling data from 12 studies, the weighted estimated prevalence of severe EPI was 8% (95% CI: 4%–14%, I^2^ = 90.96%) (Figure 4). In addition, the morbidity of severe EPI was 12% (95% CI: 7%–19%, I^2^ = 86.17%) in type 2 diabetes patients with higher insulin requirement (1 group), which was significantly higher than that in patients with lower insulin requirement (2 group) (2%, 95% CI: 0%–13%, I^2^ = 86.58%) (Supplementary Figure 2).

### 3.8. Age and EPI

Subgroup analysis based on participants' age demonstrated a higher prevalence rate in patients younger than 60 years (prevalence = 25%; 95% CI:15%–37%, I^2^ = 95.41%) compared with those older than 60 years (prevalence = 19%; 95% CI: 12%–27%, I^2^ = 72.41%) (Supplementary Figure 3).

## 4. Discussion

To the best of our knowledge, this study is the first meta-analysis of the prevalence of EPI in patients with type 2 diabetes, without pancreatitis. Our results indicate that 22% of patients with type 2 diabetes suffer from EPI. This prevalence rate is lower than previously reported by Zsóri et al. in 2018, which showed that 27% of patients with type 2 diabetes had EPI [27]. Another meta-analysis summarized the prevalence of EPI among type 2 diabetes mellitus and identified EPI in 26.2% (95% CI, 19.4%–34.3%) of 1970 type 2 diabetes patients [28]. The inclusion and exclusion criteria in both previous studies were not highly specified. Without excluding cases with pancreatitis, which may be an independent comorbidity of type 2 diabetes patients, the prevalence of EPI might be overestimated.

Several findings in our study indicate that the high prevalence of EPI in type 2 diabetes patients may be closely related to beta cell function, which determines plasma insulin levels. Firstly, our study shows that the prevalence of EPI in Asia is higher than that in Europe or Australia. Previous studies have indicated that Asian type 2 diabetes patients are more likely to be *β*-cell dysfunction related. It has been reported that insulin secretory capacity was reduced, especially in the early phase, not only in Japanese but also in other East Asians such as Korean [29, 30] and Chinese patients [31]. Yabe et al. found that type 2 diabetes in East Asians was more often characterized by *β*-cell dysfunction rather than insulin resistance [32]. A systematic review and meta-analysis of insulin response to glucose using intravenous glucose tolerance test (IVGTT) assay revealed reduced insulin secretory capacity of East Asians compared to Caucasians and Africans [33] and early studies of oral glucose tolerance test (OGTT) and IVGTT in matched cohorts of Caucasian and Japanese subjects in OGTT and IVGTT showed reduced *β*-cell function in Japanese [34, 35]. Thus, a reduced capacity of insulin secretion is typical of East Asians, which could increase the risk of EPI.

Secondly, our results showed that the prevalence of EPI in type 2 diabetes patients was correlated with insulin usage, with more insulin requirement patients with a higher risk of EPI. These results are in accordance with the findings of Hardt et al. [14] and Vujasinovic et al. [17]. In addition, a previous study has found that the C-peptide level was negatively correlated with EPI, which proved that the deficiency of insulin secretion is related to the high incidence of EPI [25, 36]. All these elements reveal insulin deficiency is associated with a higher prevalence of EPI. It has been reported that insulin has a trophic effect on pancreatic acinar tissue, and insulin deficiency might cause pancreas atrophy through the insulin-acinar portal system, which is often impaired in diabetes [36–39]. Therefore, the exocrine pancreatic function is more likely to be compromised in high insulin requirement diabetics, causing a higher risk of EPI.

Furthermore, when the prevalence of EPI with different severities was explored, it was noticed that severe pancreatic exocrine insufficiency tends to occur in patients with higher insulin requirements. The more pronounced the insulin deficiency, the greater the impairment of the exocrine function of the pancreas. We suspect that treatment with insulin in the early stage of disease may have a protective effect on exocrine pancreatic cells. Recent studies found that insulin pretreatment prevented Ca^2+^ overload, ATP depletion, inhibition of the plasma membrane Ca^2+^-ATPase, and necrosis. These data provide important evidence that insulin directly protects pancreatic acinar cell injury [39]. This may have important therapeutic implications for the treatment of EPI.

The correlation between beta cell function and insulin secretion with EPI has also been suggested by some other evidence. From the mechanism perspective, beta cell function dysfunction leads to hyperglycemic and hypoinsulinemic states, and it was demonstrated that hyperglycemia promotes proliferation and activation of pancreatic stellate cells (PSCs) and stimulates collagen production of PSCs via the protein kina-seC-p38 mitogen-activated protein kinase pathway, resulting in pancreatic fibrosis [40], while hypoinsulinemia inhibits acinar cell growth and synthesis of pancreatic enzymes [41].

In this study, we could not analyze the correlation between disease duration with EPI. Previous studies have yielded contradictory findings. Terzin et al. demonstrated no correlation between them [18], whereas a statistically significant positive correlation was determined in another two series [23, 25], which could be interpreted as diabetic microangiopathy which leads to insufficient perfusion [18]. It is well known that the incidence of microangiopathy increases with increasing diabetes period. The onset of the disease may be through local microangiopathy, resulting in ischemia of the pancreas, which could lead to pancreatic fibrosis, atrophy, and EPI [18, 42].

Due to the limitation of data from literature studies, we could not directly investigate the correlation between *β*-cell function and EPI. However, our study revealed a possible link between insulin deficiency, *β*-cell function, and EPI, which may be a novel mechanism of the disease. Previous goals of treatment aimed at repleting exocrine enzyme deficiency by oral pancreatic enzyme replacement therapy (PERT) to provide physiologic nutrition by correcting maldigestion. Our study suggests that insulin therapy with tight glycemic control might be an effective treatment for patients with type 2 diabetes and EPI. Endocrine disease therapy would potentially represent a novel therapeutic approach.

### 4.1. Study Limitations

There are several limitations to this systematic review. Firstly, the degree of heterogeneity in this study was large (I^2^  > 90%). Therefore, random-effects models were used as the most conservative approach to calculate the total effect and subgroup effect. Secondly, the sample sizes of the studies varied significantly. Nevertheless, the Cochrane handbook for systematic reviews of interventions indicated that the measurement of heterogeneity by I^2^ can be influenced by a large sample size and any amount of heterogeneity is acceptable if both accurate data and predefined eligibility criteria were provided. In addition, only a very limited number of publications have investigated the important confounding factors or subgroups (e.g., C-peptide, HbAlc, and diabetes duration) which lead to a limited analysis of risk factors. These weaknesses may have limited the generalizability of the results. Further clinical studies are needed to verify our findings.

## 5. Conclusions

In summary, this systematic review and meta-analysis lay out the prevalence and characteristics of exocrine pancreatic insufficiency in patients with type 2 diabetes. EPI had a higher prevalence in the Asian population and patients with higher insulin requirements, suggesting a mechanism related to *β*-cell function. Actively treating endocrine diseases and improving islet function may be a new way to prevent and treat pancreatic exocrine diseases.

## Figures and Tables

**Table 1:** Study and patient characteristics of the included studies.

Study	Region	Total patients (*n*)	Age, ^*∗*^y	Duration of diabetes^*∗*^	Insulin use (%)	Methods	Total EPI (*n*)	Severe EPI (*n*)
Larger 2012	Europe	472	59 (52–67)	11 (5–15)	60	FE-1	85	50
Aksoz 2020	Asia	57	51 (27–59)	12 (7–28)	57.9	FE-1	28	15
Kumar 2018	Asia	88	47.53 ± 8.9	NA	NA	FE-1	72	30
Čabarkapa 2018	Europe	62	61.1 ± 8.9	16.1 ± 8.9	NA	FE-1	13	3
Hardt 2003	Europe	697	53.8 (21–78)	8.7 (<1–39)	52.5	FE-1	247	139
Shivaprasad 2015	Asia	95	50.2 ± 10.2	NA	NA	FE-1	28	3
Søfteland 2019	Europe	52	NA	NA	NA	FE-1	3	NA
Terzin 2014	Europe	101	60.9 ± 11.2	11.2 ± 8.8	61.4	FE-1	17	3
Vujasinovic 2013	Europe	100	NA	NA	50	FE-1	5	3
Nunes 2003	Europe	42	62 ± 10	11.5 ± 8	28.6	FE-1	15	9
Riceman 2019	Australia	109	40–80	NA	NA	FE-1	10	4
Lv 2021	Asia	85	61.4 ± 12.3	7 (7)	49.4	FE-1	16	3
Anoop 2021	Asia	118	56.1 ± 8.4	12.3 ± 6.2	22.9	72 h FF	53	NA

^*∗*^Data are expressed as median, mean (SD), or range. NA: not applicable; 72 h FF: 72-hours fecal fat.

## Data Availability

All the data generated or analyzed during this study are included in this published article and supplementary information files.

## Supplementary Materials

Supplementary Materials Supplementary Table 1: the Joanna Briggs Institute Prevalence Critical Appraisal Tool was used to evaluate the quality of selected articles. Supplementary Figure 1: the figure reveals the relationship between the prevalence of EPI and insulin use in patients with type 2 diabetes. Supplementary Figure 2: the figure reveals the relationship between the prevalence of severe EPI and insulin use in patients with type 2 diabetes. Supplementary Figure 3: the figure shows the association between EPI prevalence and patients' age. Supplementary Table 1: Joanna Briggs Institute's critical appraisal checklist for studies reporting prevalence data. Figure 1: insulin use and EPI. Figure 2: insulin use and severity of EPI. Figure 3: age and EPI.
