# Supplementary material for: The Prevalence and Characteristics of Exocrine Pancreatic Insufficiency in Patients with Type 2 Diabetes: A Systematic Review and Meta-Analysis
Source: Int J Endocrinol. 2022 Jul 19;2022:7764963. doi: 10.1155/2022/7764963 (PMC9536940; doi:10.1155/2022/7764963)
Supplement: Supplementary Materials — Supplementary Table 1: the Joanna Briggs Institute Prevalence Critical Appraisal Tool was used to evaluate the quality of selected articles. Supplementary Figure 1: the figure reveals the relationship between the prevalence of EPI and insulin use in patients with type 2 diabetes. Supplementary Figure 2: the figure reveals the relationship between the prevalence of severe EPI and insulin use in patients with type 2 diabetes. Supplementary Figure 3: the figure shows the association between EPI prevalence and patients' age. Supplementary Table 1: Joanna Briggs Institute's critical appraisal checklist for studies reporting prevalence data. Figure 1: insulin use and EPI. Figure 2: insulin use and severity of EPI. Figure 3: age and EPI. [file 7764963.f1.zip › Supplementary Table1 Joanna Briggs Institute's critical appraisal checklist for studies reporting prevalence data.docx]

| Supplementary Table1 Joanna Briggs Institute’s critical appraisal checklist for studies reporting prevalence data | | | | | | | | | | | | | |
| --- | --- | --- | --- | --- | --- | --- | --- | --- | --- | --- | --- | --- | --- |
|  | Larger  2012 | Aksoz  2020 | Prasanna  2018 | Čabarkapa  2018 | Hardt  2003 | Shivaprasad  2015 | Søfteland  2019 | Terzin  2014 | Vujasinovic  2013 | Nunes  2003 | Lv  2021 | Annop  2021 | Riceman  2019 |
| Was the sample representative of  the target population? | Yes | Yes | Yes | Yes | Yes | Yes | Yes | Yes | Yes | Yes | Yes | Yes | Yes |
| Were study participants recruited in an appropriate way? | No | Unclear | No | Unclear | Unclear | Unclear | Yes | Yes | Yes | Unclear | Yes | Unclear | Yes |
| Was the sample size adequate? | Yes | Yes | Yes | Yes | Yes | Yes | Yes | Yes | Yes | Yes | Yes | Yes | Yes |
| Were the study subjects and the setting described in detail? | Yes | Not applicable | Yes | Yes | Yes | Yes | Yes | Yes | Yes | Not applicable | Yes | Yes | Yes |
| Was the data analysis conducted with sufficient coverage of the identified sample? | Yes | Yes | Yes | Yes | Yes | Yes | Yes | Yes | Yes | Yes | Yes | Yes | Yes |
| Were objective, standard criteria used for the measurement of the condition? | Yes | Yes | Yes | Yes | Yes | Yes | Yes | Yes | Yes | Yes | Yes | Yes | Yes |
| Was the condition measured reliably? | Yes | Yes | Yes | Yes | Yes | Yes | Yes | Yes | Yes | Yes | Yes | Yes | Yes |
| Was there appropriate statistical analysis? | Yes | Yes | Yes | Yes | Yes | Yes | Yes | Yes | Yes | Yes | Yes | Yes | Yes |
| Are all important confounding factors/subgroups/differences identified and accounted for? | Yes | Yes | Yes | Yes | Yes | Yes | No | Yes | No | No | Yes | Yes | No |
| Were subpopulations identified using objective criteria? | No | No | No | No | No | No | No | Yes | No | No | No | No | No |
| A total | 8 | 7 | 9 | 8 | 8 | 8 | 8 | 10 | 8 | 6 | 9 | 8 | 8 |
