# Supplementary figures and images for: The Prevalence and Characteristics of Exocrine Pancreatic Insufficiency in Patients with Type 2 Diabetes: A Systematic Review and Meta-Analysis
Source: Int J Endocrinol. 2022 Jul 19;2022:7764963. doi: 10.1155/2022/7764963 (PMC9536940; doi:10.1155/2022/7764963)

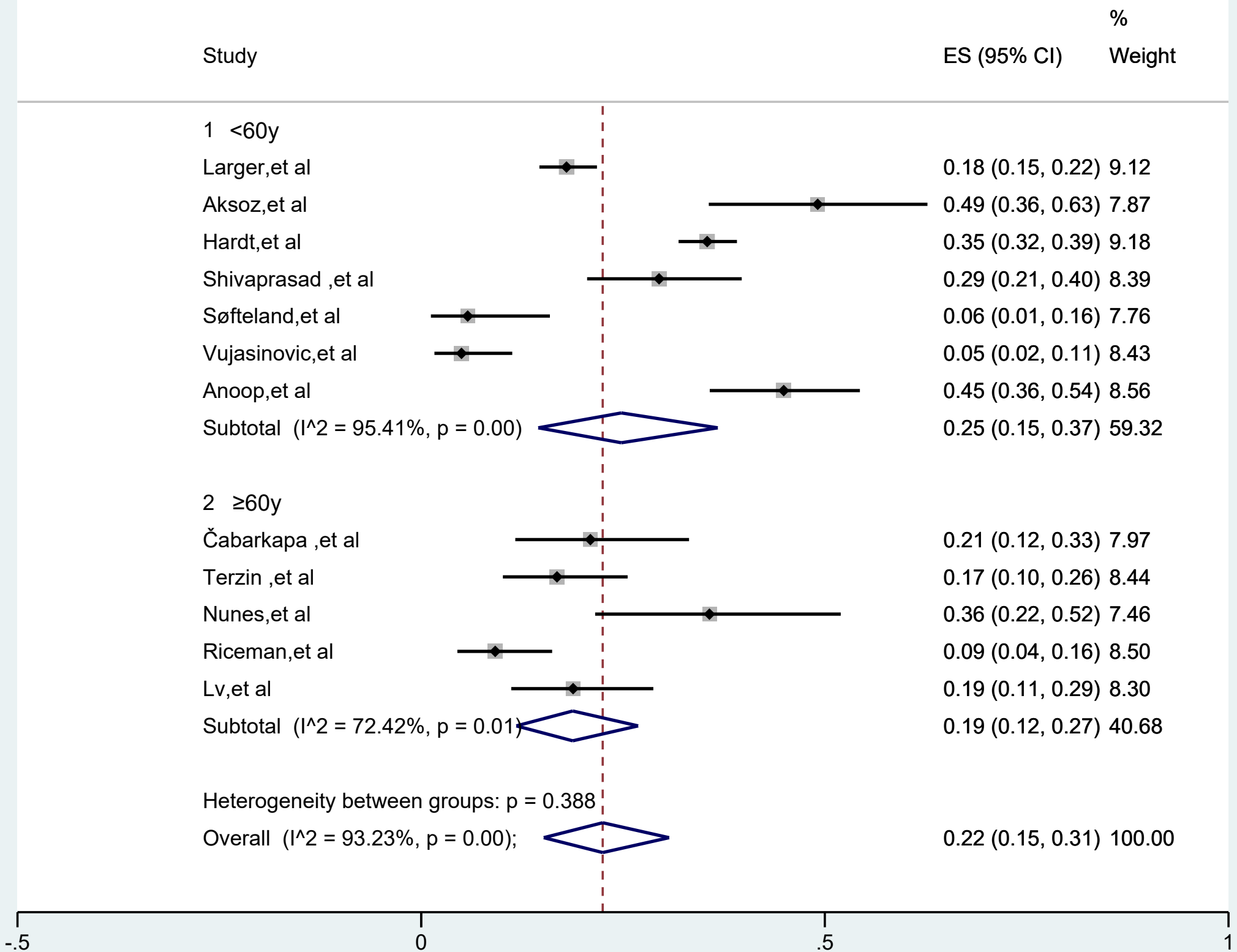

Supplement: Supplementary Materials — Supplementary Table 1: the Joanna Briggs Institute Prevalence Critical Appraisal Tool was used to evaluate the quality of selected articles. Supplementary Figure 1: the figure reveals the relationship between the prevalence of EPI and insulin use in patients with type 2 diabetes. Supplementary Figure 2: the figure reveals the relationship between the prevalence of severe EPI and insulin use in patients with type 2 diabetes. Supplementary Figure 3: the figure shows the association between EPI prevalence and patients' age. Supplementary Table 1: Joanna Briggs Institute's critical appraisal checklist for studies reporting prevalence data. Figure 1: insulin use and EPI. Figure 2: insulin use and severity of EPI. Figure 3: age and EPI. [file 7764963.f1.zip › Supplementary Fig 3. Age and EPI.pdf]

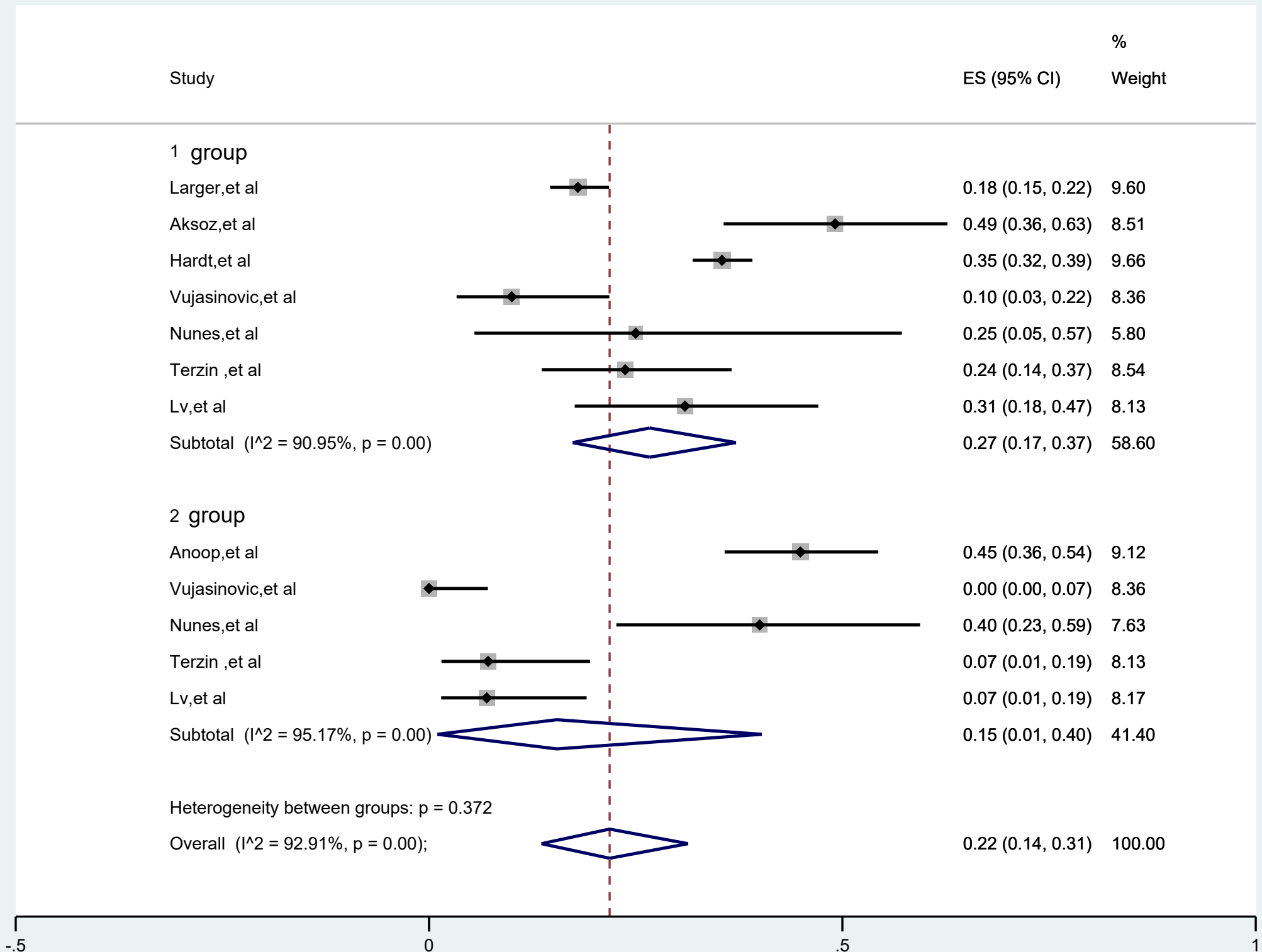

Supplement: Supplementary Materials — Supplementary Table 1: the Joanna Briggs Institute Prevalence Critical Appraisal Tool was used to evaluate the quality of selected articles. Supplementary Figure 1: the figure reveals the relationship between the prevalence of EPI and insulin use in patients with type 2 diabetes. Supplementary Figure 2: the figure reveals the relationship between the prevalence of severe EPI and insulin use in patients with type 2 diabetes. Supplementary Figure 3: the figure shows the association between EPI prevalence and patients' age. Supplementary Table 1: Joanna Briggs Institute's critical appraisal checklist for studies reporting prevalence data. Figure 1: insulin use and EPI. Figure 2: insulin use and severity of EPI. Figure 3: age and EPI. [file 7764963.f1.zip › Supplementary Fig. 1 Insulin use and EPI.pdf]

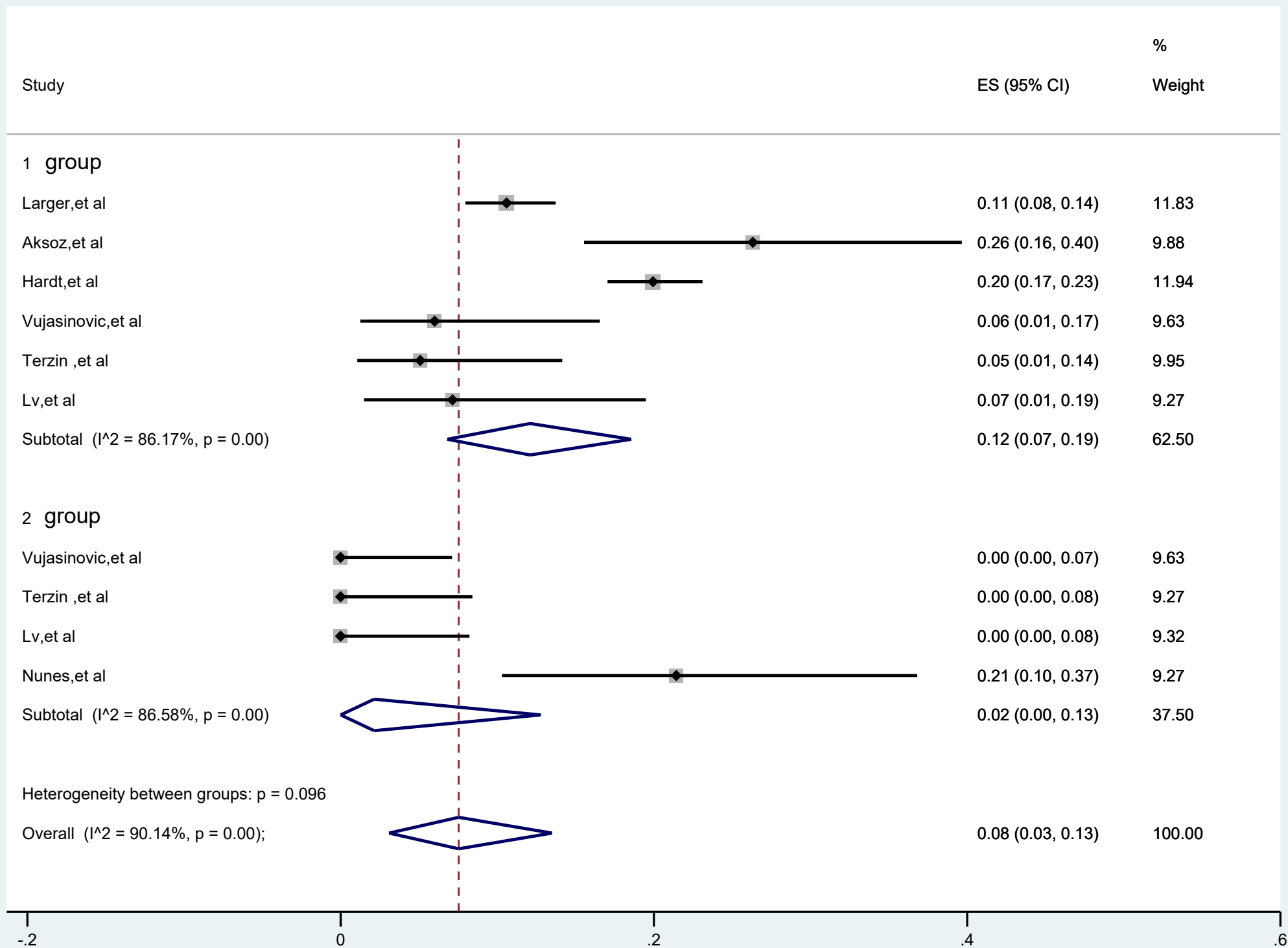

Supplement: Supplementary Materials — Supplementary Table 1: the Joanna Briggs Institute Prevalence Critical Appraisal Tool was used to evaluate the quality of selected articles. Supplementary Figure 1: the figure reveals the relationship between the prevalence of EPI and insulin use in patients with type 2 diabetes. Supplementary Figure 2: the figure reveals the relationship between the prevalence of severe EPI and insulin use in patients with type 2 diabetes. Supplementary Figure 3: the figure shows the association between EPI prevalence and patients' age. Supplementary Table 1: Joanna Briggs Institute's critical appraisal checklist for studies reporting prevalence data. Figure 1: insulin use and EPI. Figure 2: insulin use and severity of EPI. Figure 3: age and EPI. [file 7764963.f1.zip › Supplementary Fig. 2 Insulin use and severity of EPI.pdf]
